# Supplementary material for: Assessing distinct patterns of cognitive aging using tissue-specific brain age prediction based on diffusion tensor imaging and brain morphometry
Source: PeerJ. 2018 Nov 30;6:e5908. doi: 10.7717/peerj.5908 (PMC6276592; doi:10.7717/peerj.5908)
Supplement: Table S1 — * FDR significant ** Bonferroni significant. MoCA: Montreal Cognitive Assessment. WASI: Wechsler Abbreviated Scale of Intelligence. CVLT: California Verbal Learning Test. STROOP: Delis-Kaplan Executive Function System (D-KEFS) color word interference test. CP: Cognitive Assessment at Bedside for iPAD (CabPAD). WM: working memory. TVA: Theory of Visual Attention. ls: longest serie. ss: sum scores. tot: total. [file peerj-06-5908-s001.docx]

| Test | Adj R^2^ no-BAG | BAG | Main effect Age  *t(p)* | Main effect Age^2^  *t(p)* | Main effect Sex  *t(p)* | Main effect BAG  *t(p)* | Adj R^2^ |
| --- | --- | --- | --- | --- | --- | --- | --- |
| MoCA | 0.088 | T1 | -4.5563 (<0.001) | -0.2752 (0.7834) | -2.1792 (0.0302) | -0.1146 (0.9088) | 0.0845 |
|  |  | DTI | -4.5575 (<0.001) | -0.3078 (0.7585) | -2.1727 (0.0307) | 1.5937 (0.1122) | 0.0934 |
|  |  | Combined | -4.561 (<0.001) | -0.2413 (0.8095) | -2.1894 (0.0295) | -0.4399 (0.6604) | 0.0851 |
| WASI words | 0.0792 | T1 | 4.7079 (<0.001) | -1.5614 (0.1197) | 0.4698 (0.6389) | -0.1719 (0.8637) | 0.0756 |
|  |  | DTI | 4.7017 (<0.001) | -1.5532 (0.1216) | 0.4775 (0.6334) | -0.7843 (0.4336) | 0.0778 |
|  |  | Combined | 4.7058 (<0.001) | -1.5325 (0.1266) | 0.4655 (0.642) | -0.3554 (0.7226) | 0.076 |
| WASI matrix | 0.1825 | T1 | -7.6085 (<0.001) | -1.3223 (0.1873) | 0.048 (0.9618) | -0.8787 (0.3804) | 0.1817 |
|  |  | DTI | -7.6627 (<0.001) | -1.311 (0.1911) | 0.0606 (0.9517) | -1.6227 (0.1059) | 0.1876 |
|  |  | Combined | -7.6132 (<0.001) | -1.2678 (0.206) | 0.0407 (0.9676) | -1.0117 (0.3126) | 0.1825 |
| CVLT learning 1-5 | 0.1757 | T1 | -5.0181 (<0.001) | 0.5198 (0.6036) | -5.2162 (<0.001) | -0.2662 (0.7903) | 0.1727 |
|  |  | DTI | -5.0228 (<0.001) | 0.5204 (0.6032) | -5.2181 (<0.001) | -0.3723 (0.71) | 0.1729 |
|  |  | Combined | -5.0196 (<0.001) | 0.5363 (0.5922) | -5.2205 (<0.001) | -0.2959 (0.7675) | 0.1727 |
| CVLT interference | 0.0593 | T1 | -4.3175 (<0.001) | -0.062 (0.9506) | -0.3788 (0.7051) | -0.9545 (0.3407) | 0.0589 |
|  |  | DTI | -4.3143 (<0.001) | -0.086 (0.9316) | -0.3772 (0.7064) | -0.2366 (0.8132) | 0.0558 |
|  |  | Combined | -4.3124 (<0.001) | -0.075 (0.9403) | -0.3794 (0.7047) | -0.1796 (0.8576) | 0.0557 |
| CVLT recall | 0.2401 | T1 | -6.4676 (<0.001) | 0.6091 (0.543) | -5.8925 (<0.001) | -0.5048 (0.6141) | 0.2379 |
|  |  | DTI | -6.4667 (<0.001) | 0.5965 (0.5514) | -5.8892 (<0.001) | -0.1381 (0.8902) | 0.2371 |
|  |  | Combined | -6.4859 (<0.001) | 0.6973 (0.4863) | -5.9255 (<0.001) | -1.1682 (0.2438) | 0.2412 |
| CVLT delayed recall | 0.1822 | T1 | -4.9433 (<0.001) | 0.6624 (0.5083) | -5.4919 (<0.001) | 0.1193 (0.9052) | 0.179 |
|  |  | DTI | -4.9411 (<0.001) | 0.6618 (0.5087) | -5.4912 (<0.001) | 0.2079 (0.8354) | 0.1791 |
|  |  | Combined | -4.9454 (<0.001) | 0.6981 (0.4857) | -5.4986 (<0.001) | -0.3668 (0.7141) | 0.1794 |
| CVLT recognition hits | 0.0464 | T1 | -2.615 (0.0095) | -0.3694 (0.7121) | -2.5144 (0.0125) | -0.8444 (0.3992) | 0.0453 |
|  |  | DTI | -2.6175 (0.0094) | -0.3994 (0.6899) | -2.504 (0.0129) | 0.1039 (0.9173) | 0.0427 |
|  |  | Combined | -2.622 (0.0093) | -0.3004 (0.7641) | -2.5328 (0.0119) | -1.0382 (0.3002) | 0.0467 |
| CVLT recognition errors | 0.1567 | T1 | 5.2609 (<0.001) | 1.4118 (0.1592) | 3.7395 (<0.001) | -0.8964 (0.3709) | 0.1561 |
|  |  | DTI | 5.2485 (<0.001) | 1.3947 (0.1643) | 3.7336 (<0.001) | -0.5993 (0.5495) | 0.1546 |
|  |  | Combined | 5.2514 (<0.001) | 1.4099 (0.1598) | 3.7277 (<0.001) | -0.3837 (0.7015) | 0.1539 |
| CVLT recog misses | 0.0464 | T1 | 2.615 (0.0095) | 0.3694 (0.7121) | 2.5144 (0.0125) | 0.8444 (0.3992) | 0.0453 |
|  |  | DTI | 2.6175 (0.0094) | 0.3994 (0.6899) | 2.504 (0.0129) | -0.1039 (0.9173) | 0.0427 |
|  |  | Combined | 2.622 (0.0093) | 0.3004 (0.7641) | 2.5328 (0.0119) | 1.0382 (0.3002) | 0.0467 |
| CVLT recog false alarm | 0.1191 | T1 | 4.4883 (<0.001) | 1.4053 (0.1612) | 3.1557 (0.0018) | -0.8236 (0.411) | 0.1179 |
|  |  | DTI | 4.4728 (<0.001) | 1.3906 (0.1656) | 3.1567 (0.0018) | -0.5544 (0.5798) | 0.1167 |
|  |  | Combined | 4.4784 (<0.001) | 1.4159 (0.158) | 3.1475 (0.0018) | -0.4786 (0.6326) | 0.1164 |
| CVLT recog correct rejection | 0.1567 | T1 | -5.2609 (<0.001) | -1.4118 (0.1592) | -3.7395 (<0.001) | 0.8964 (0.3709) | 0.1561 |
|  |  | DTI | -5.2485 (<0.001) | -1.3947 (0.1643) | -3.7336 (<0.001) | 0.5993 (0.5495) | 0.1546 |
|  |  | Combined | -5.2514 (<0.001) | -1.4099 (0.1598) | -3.7277 (<0.001) | 0.3837 (0.7015) | 0.1539 |
| CVLT d’ | 0.1548 | T1 | -5.0157 (<0.001) | -0.7018 (0.4835) | -4.1938 (<0.001) | 0.3855 (0.7002) | 0.1519 |
|  |  | DTI | -5.0104 (<0.001) | -0.7106 (0.478) | -4.1973 (<0.001) | 0.8695 (0.3854) | 0.1539 |
|  |  | Combined | -5.0122 (<0.001) | -0.7079 (0.4796) | -4.1897 (<0.001) | 0.2343 (0.8149) | 0.1516 |
| STROOP 1 | 0.1181 | T1 | 5.2178 (<0.001) | 1.8817 (0.061) | 1.9786 (0.0489) | 2.6477 (0.0086) | 0.1385 |
|  |  | DTI | 5.1698 (<0.001) | 1.915 (0.0566) | 1.9488 (0.0524) | 1.6361 (0.103) | 0.1239 |
|  |  | Combined | 5.2707 (<0.001) | 1.7146 (0.0876) | 2.0444 (0.0419) | 3.243 (0.0013) | 0.1499 |
| STROOP 2 | 0.0565 | T1 | 2.955 (0.0034) | 1.8769 (0.0617) | 1.7273 (0.0853) | 0.0898 (0.9286) | 0.0528 |
|  |  | DTI | 2.9455 (0.0035) | 1.897 (0.059) | 1.7093 (0.0886) | -0.5228 (0.6016) | 0.0538 |
|  |  | Combined | 2.9595 (0.0034) | 1.8439 (0.0664) | 1.7389 (0.0833) | 0.3307 (0.7411) | 0.0531 |
| STROOP 3 | 0.2519 | T1 | 7.9387 (<0.001) | 4.0215 (<0.001)** | 2.0391 (0.0425) | 1.4584 (0.146) | 0.2552 |
|  |  | DTI | 8.0033 (<0.001) | 4.0506 (<0.001)** | 2.0666 (0.0398) | 2.245 (0.0256) | 0.2635 |
|  |  | Combined | 8.0064 (<0.001) | 3.9001 (<0.001)** | 2.0915 (0.0375) | 2.3542 (0.0193) | 0.265 |
| STROOP 4 | 0.2092 | T1 | 7.727 (<0.001) | 2.7144 (0.0071) | 1.1078 (0.269) | 1.1959 (0.2329) | 0.2105 |
|  |  | DTI | 7.7745 (<0.001) | 2.7322 (0.0067) | 1.1277 (0.2605) | 1.7316 (0.0846) | 0.2154 |
|  |  | Combined | 7.8094 (<0.001) | 2.5945 (0.01) | 1.1659 (0.2448) | 2.2006 (0.0287) | 0.221 |
| STROOP mean 1 and 2 | 0.1065 | T1 | 4.6014 (<0.001) | 2.1809 (0.0301) | 1.8851 (0.0606) | 1.5137 (0.1314) | 0.1111 |
|  |  | DTI | 4.5693 (<0.001) | 2.2194 (0.0274) | 1.8487 (0.0657) | 0.3205 (0.7489) | 0.1033 |
|  |  | Combined | 4.6255 (<0.001) | 2.0629 (0.0402) | 1.9411 (0.0534) | 1.8349 (0.0677) | 0.1148 |
| STROOP 3 minus mean 1 and 2 | 0.2486 | T1 | 7.6928 (<0.001) | 4.0734 (<0.001)** | 2.0223 (0.0442) | 1.0347 (0.3018) | 0.2488 |
|  |  | DTI | 7.7161 (<0.001) | 4.0759 (<0.001)** | 2.0485 (0.0416) | 1.2741 (0.2038) | 0.2504 |
|  |  | Combined | 7.7517 (<0.001) | 3.9419 (<0.001)** | 2.0994 (0.0368) | 1.8804 (0.0612) | 0.2561 |
| STROOP 4 minus mean 1 and 2 | 0.2225 | T1 | 7.7912 (<0.001) | 3.224 (0.0014)* | 1.0274 (0.3052) | 0.7875 (0.4317) | 0.2213 |
|  |  | DTI | 7.7853 (<0.001) | 3.233 (0.0014)* | 1.0273 (0.3053) | 0.5573 (0.5778) | 0.2203 |
|  |  | Combined | 7.8437 (<0.001) | 3.1135 (0.0021)* | 1.0945 (0.2748) | 1.4951 (0.1362) | 0.2263 |
| CP - Right motor speed | 0.3621 | T1 | -12.2583 (<0.001) | 0.4241 (0.6718) | -0.4499 (0.6532) | -1.56 (0.12) | 0.3656 |
|  |  | DTI | -12.201 (<0.001) | 0.3813 (0.7033) | -0.4415 (0.6592) | -0.3491 (0.7273) | 0.3599 |
|  |  | Combined | -12.2818 (<0.001) | 0.5259 (0.5994) | -0.4741 (0.6358) | -1.8479 (0.0658) | 0.368 |
| CP - Left motor speed | 0.3586 | T1 | -12.1901 (<0.001) | -1.3758 (0.1701) | 0.5329 (0.5946) | -1.9551 (0.0517) | 0.3656 |
|  |  | DTI | -12.1163 (<0.001) | -1.4124 (0.159) | 0.5396 (0.5899) | -0.8474 (0.3976) | 0.3579 |
|  |  | Combined | -12.2849 (<0.001) | -1.2158 (0.2252) | 0.4997 (0.6177) | -2.7995 (0.0055) | 0.3752 |
| CP - FAS semantic flow | 0.1047 | T1 | -3.0413 (0.0026) | -2.5748 (0.0106) | -3.2544 (0.0013) | -2.0265 (0.0437) | 0.1153 |
|  |  | DTI | -3.0473 (0.0025) | -2.6035 (0.0098) | -3.2424 (0.0013) | -2.0796 (0.0385) | 0.116 |
|  |  | Combined | -3.0316 (0.0027) | -2.4889 (0.0134) | -3.2639 (0.0012) | -1.642 (0.1018) | 0.1105 |
| CP - Visual WM forward ls | 0.0897 | T1 | -5.3048 (<0.001) | -0.2803 (0.7795) | 0.3424 (0.7323) | -0.5645 (0.5729) | 0.0873 |
|  |  | DTI | -5.3364 (<0.001) | -0.2747 (0.7838) | 0.352 (0.7252) | -1.71 (0.0885) | 0.0966 |
|  |  | Combined | -5.3039 (<0.001) | -0.2873 (0.7742) | 0.3445 (0.7307) | -0.2836 (0.7769) | 0.0864 |
| CP - Visual WM forward ss | 0.1388 | T1 | -6.5947 (<0.001) | -0.6439 (0.5202) | -0.0916 (0.9271) | -0.1756 (0.8608) | 0.1355 |
|  |  | DTI | -6.6142 (<0.001) | -0.6289 (0.53) | -0.0903 (0.9281) | -1.1522 (0.2503) | 0.1399 |
|  |  | Combined | -6.5945 (<0.001) | -0.657 (0.5118) | -0.0873 (0.9305) | 0.0421 (0.9664) | 0.1354 |
| CP - Visual WM backward ls | 0.0852 | T1 | -4.6076 (<0.001) | -0.9343 (0.351) | -1.567 (0.1184) | -0.068 (0.9458) | 0.0816 |
|  |  | DTI | -4.6299 (<0.001) | -0.9132 (0.362) | -1.5757 (0.1163) | -1.3135 (0.1902) | 0.0878 |
|  |  | Combined | -4.6163 (<0.001) | -0.8736 (0.3831) | -1.5842 (0.1144) | -0.7237 (0.4699) | 0.0835 |
| CP - Visual WM backward ss | 0.1026 | T1 | -5.4768 (<0.001) | -0.619 (0.5365) | -0.8352 (0.4044) | -0.2473 (0.8049) | 0.0993 |
|  |  | DTI | -5.4994 (<0.001) | -0.604 (0.5464) | -0.8389 (0.4023) | -1.2897 (0.1983) | 0.105 |
|  |  | Combined | -5.4903 (<0.001) | -0.5466 (0.5852) | -0.8555 (0.3931) | -0.9558 (0.3401) | 0.1023 |
| CP - Visual WM ss | 0.1618 | T1 | -7.0453 (<0.001) | -0.9494 (0.3433) | -0.6911 (0.4902) | -0.2639 (0.7921) | 0.1588 |
|  |  | DTI | -7.0749 (<0.001) | -0.9354 (0.3504) | -0.6944 (0.4881) | -1.3432 (0.1804) | 0.1645 |
|  |  | Combined | -7.051 (<0.001) | -0.9076 (0.365) | -0.7016 (0.4836) | -0.5866 (0.558) | 0.1597 |
| CP - Spatial stroop congruent | 0.2152 | T1 | 8.6103 (<0.001) | 0.5061 (0.6132) | -1.099 (0.2728) | 2.1677 (0.0311) | 0.2265 |
|  |  | DTI | 8.6646 (<0.001) | 0.539 (0.5904) | -1.1012 (0.2719) | 2.6844 (0.0078) | 0.234 |
|  |  | Combined | 8.8148 (<0.001) | 0.27 (0.7874) | -1.0172 (0.3101) | 3.8562 (<0.001)** | 0.2561 |
| CP - Spatial stroop incongruent | 0.2498 | T1 | 9.5215 (<0.001) | -0.3129 (0.7546) | -0.6399 (0.5228) | 2.6625 (0.0083) | 0.2674 |
|  |  | DTI | 9.5663 (<0.001) | -0.2497 (0.803) | -0.6711 (0.5028) | 2.8797 (0.0043) | 0.2708 |
|  |  | Combined | 9.6908 (<0.001) | -0.5285 (0.5976) | -0.5807 (0.5619) | 3.8325 (<0.001)* | 0.2883 |
| CP - Spatial stroop numb of reps | 0.2613 | T1 | -9.7578 (<0.001) | -0.1542 (0.8776) | 1.2196 (0.2237) | -2.2091 (0.0281) | 0.2725 |
|  |  | DTI | -9.8334 (<0.001) | -0.1782 (0.8587) | 1.2369 (0.2173) | -2.951 (0.0035) | 0.2831 |
|  |  | Combined | -9.967 (<0.001) | 0.0905 (0.928) | 1.1582 (0.2479) | -3.8672 (<0.001)** | 0.2999 |
| CP - Spatial stroop incong - cong | 0.1076 | T1 | 5.7387 (<0.001) | -0.9619 (0.337) | -0.4011 (0.6887) | 1.5968 (0.1116) | 0.1131 |
|  |  | DTI | 5.7189 (<0.001) | -0.9105 (0.3635) | -0.4223 (0.6732) | 0.9815 (0.3273) | 0.1075 |
|  |  | Combined | 5.7304 (<0.001) | -1.0014 (0.3176) | -0.3888 (0.6978) | 1.2835 (0.2005) | 0.1099 |
| CP - Spatspan ls | 0.3027 | T1 | -9.0803 (<0.001) | 0.8225 (0.4116) | -4.9183 (<0.001) | -0.0628 (0.95) | 0.3 |
|  |  | DTI | -9.1511 (<0.001) | 0.8555 (0.3931) | -4.9703 (<0.001) | -1.5904 (0.113) | 0.307 |
|  |  | Combined | -9.0818 (<0.001) | 0.8301 (0.4073) | -4.9202 (<0.001) | -0.1448 (0.885) | 0.3 |
| CP - Spatspan total | 0.3033 | T1 | -9.2413 (<0.001) | 0.5919 (0.5545) | -4.6451 (<0.001) | 0.085 (0.9324) | 0.3006 |
|  |  | DTI | -9.3007 (<0.001) | 0.6245 (0.5329) | -4.6899 (<0.001) | -1.3875 (0.1665) | 0.3059 |
|  |  | Combined | -9.2443 (<0.001) | 0.6028 (0.5472) | -4.6491 (<0.001) | -0.1118 (0.9111) | 0.3006 |
| CP - Coding corr | 0.5307 | T1 | -16.7381 (<0.001) | -0.3279 (0.7433) | -2.3396 (0.0201) | -1.596 (0.1117) | 0.5335 |
|  |  | DTI | -17.0629 (<0.001) | -0.3481 (0.7281) | -2.3804 (0.018) | -3.3875 (<0.001)* | 0.5495 |
|  |  | Combined | -16.9736 (<0.001) | -0.1442 (0.8855) | -2.4411 (0.0153) | -2.976 (0.0032) | 0.5449 |
| TVA - Short-term memory storage (*K*) | 0.1941 | T1 | -7.7533 (<0.001) | -0.0891 (0.9291) | -1.4529 (0.1475) | -1.1129 (0.2668) | 0.1949 |
|  |  | DTI | -7.7958 (<0.001) | -0.0894 (0.9288) | -1.471 (0.1426) | -2.0247 (0.044) | 0.2039 |
|  |  | Combined | -7.7352 (<0.001) | -0.0343 (0.9726) | -1.4655 (0.144) | -0.9449 (0.3456) | 0.1938 |
| TVA - Perceptual threshold (*t_0_*) | 0.1146 | T1 | 5.7716 (<0.001) | 0.9866 (0.3248) | -2.1312 (0.034) | 0.9264 (0.3551) | 0.1141 |
|  |  | DTI | 5.7764 (<0.001) | 1.0081 (0.3144) | -2.1342 (0.0338) | 1.0951 (0.2745) | 0.1153 |
|  |  | Combined | 5.784 (<0.001) | 0.8478 (0.3973) | -2.1074 (0.0361) | 1.7412 (0.0829) | 0.1217 |
| TVA - Processing speed (*C*) | 0.0699 | T1 | -4.66 (<0.001) | 0.1122 (0.9107) | 0.3556 (0.7225) | 0.804 (0.4222) | 0.0686 |
|  |  | DTI | -4.6708 (<0.001) | 0.135 (0.8927) | 0.3579 (0.7207) | 0.1387 (0.8898) | 0.0662 |
|  |  | Combined | -4.6734 (<0.001) | 0.0619 (0.9507) | 0.3657 (0.7149) | 0.8815 (0.3789) | 0.0691 |
| Cluster 1 | 0.2446 | T1 | -7.1703 (<0.001) | -0.4829 (0.6296) | -4.8899 (<0.001) | -0.1702 (0.865) | 0.2416 |
|  |  | DTI | -7.1589 (<0.001) | -0.5024 (0.6158) | -4.8709 (<0.001) | 0.3825 (0.7024) | 0.242 |
|  |  | Combined | -7.1752 (<0.001) | -0.4542 (0.6501) | -4.8989 (<0.001) | -0.3391 (0.7349) | 0.2419 |
| Cluster 2 | 0.1695 | T1 | -7.2665 (<0.001) | -0.5147 (0.6072) | 1.6797 (0.0943) | -0.0814 (0.9352) | 0.1662 |
|  |  | DTI | -7.2772 (<0.001) | -0.5154 (0.6068) | 1.6829 (0.0937) | -0.6514 (0.5154) | 0.1676 |
|  |  | Combined | -7.2707 (<0.001) | -0.4611 (0.6451) | 1.6725 (0.0957) | -0.5914 (0.5548) | 0.1674 |
| Cluster 3 | 0.0767 | T1 | -2.065 (0.0399) | -1.6934 (0.0916) | -3.4589 (<0.001) | -0.7735 (0.4399) | 0.0753 |
|  |  | DTI | -2.0812 (0.0384) | -1.693 (0.0917) | -3.4728 (<0.001) | -1.8207 (0.0698) | 0.0849 |
|  |  | Combined | -2.0636 (0.0401) | -1.6405 (0.1021) | -3.4746 (<0.001) | -0.848 (0.3972) | 0.0757 |
| Cluster 4 | 0.2757 | T1 | -10.1014 (<0.001) | 0.3766 (0.7068) | 1.098 (0.2733) | -2.5518 (0.0113) | 0.2912 |
|  |  | DTI | -10.118 (<0.001) | 0.31 (0.7568) | 1.1208 (0.2635) | -2.5199 (0.0124) | 0.2908 |
|  |  | Combined | -10.2703 (<0.001) | 0.5794 (0.5629) | 1.0357 (0.3014) | -3.6468 (<0.001)* | 0.3095 |
| Cluster 5 | 0.2124 | T1 | -7.1604 (<0.001) | -3.4563 (<0.001)* | -1.6891 (0.0924) | -1.0197 (0.3089) | 0.2125 |
|  |  | DTI | -7.1405 (<0.001) | -3.4709 (<0.001)* | -1.6759 (0.095) | -0.5011 (0.6167) | 0.21 |
|  |  | Combined | -7.2104 (<0.001) | -3.3405 (0.001)* | -1.7586 (0.0799) | -1.6393 (0.1024) | 0.2177 |
| Cluster 6 | 0.51 | T1 | -15.9382 (<0.001) | -1.0984 (0.2732) | -0.8171 (0.4147) | -1.8518 (0.0653) | 0.5149 |
|  |  | DTI | -15.9778 (<0.001) | -1.1226 (0.2627) | -0.8054 (0.4214) | -2.0589 (0.0406) | 0.5165 |
|  |  | Combined | -16.0092 (<0.001) | -0.9591 (0.3385) | -0.8532 (0.3944) | -2.3608 (0.019) | 0.5191 |
| Cluster 7 | 0.1381 | T1 | -6.5016 (<0.001) | -0.6594 (0.5102) | -0.5961 (0.5516) | -0.3018 (0.7631) | 0.135 |
|  |  | DTI | -6.5363 (<0.001) | -0.643 (0.5208) | -0.5958 (0.5518) | -1.5824 (0.1148) | 0.1432 |
|  |  | Combined | -6.5065 (<0.001) | -0.6229 (0.5339) | -0.6062 (0.5449) | -0.5676 (0.5708) | 0.1358 |

**Table S1.** Cognitive associations with Brain Age Gap (BAG) using non-linear models, including age, age^2^ and sex as covariates – statistics. * FDR significant ** Bonferroni significant. MoCA: Montreal Cognitive Assessment. WASI: Wechsler Abbreviated Scale of Intelligence. CVLT: California Verbal Learning Test. STROOP: Delis-Kaplan Executive Function System (D-KEFS) color word interference test. CP: Cognitive Assessment at Bedside for iPAD (CabPAD). WM: working memory. TVA: Theory of Visual Attention. ls: longest serie. ss: sum scores. tot: total.
