# Supplementary material for: Assessing distinct patterns of cognitive aging using tissue-specific brain age prediction based on diffusion tensor imaging and brain morphometry
Source: PeerJ. 2018 Nov 30;6:e5908. doi: 10.7717/peerj.5908 (PMC6276592; doi:10.7717/peerj.5908)
Supplement: Table S2 — WASI= Wechsler Abbreviated Scale of Intelligence, CLVT= California Verbal Learning Test, STROOP= Delis-Kaplan Executive Function System (D-KEFS) color word interference test, CP= Cognitive Assessment at Bedside for iPAD (CabPAD), TVA= Theory of Visual Attention. [file peerj-06-5908-s002.docx]

| Abbreviations | Descriptions |
| --- | --- |
| MoCA | Montreal Cognitive Assessment |
| WASI Word | WASI – Vocabulary subscale |
| WASI Matrix | WASI – Matrix Reasoning subscale |
| CVLT SUM 1 5 | CVLT- sum of raw scores learning 1-5 |
| CVLT Interf | CVLT interference |
| CVLT Um Recall | CVLT immediate recall |
| CVLT Delayed R | CVLT delayed recall |
| CVLT Rec Hit | CVLT recognition hit |
| CVLT cr | CVLT recognition correct rejection |
| CVLT Rec Error | CVLT recognition errors total |
| CVLT miss | CVLT recognition misses (omissions) |
| CVLT fa | CVLT recognition false alarm (commissions) |
| CVLT dprime | CVLT d-prime |
| Stroop1 time | STROOP 1 color naming (time) |
| Stroop2 time | STROOP 2 reading (time) |
| Stroop3 time | STROOP 3 inhibition (time) |
| Stroop4 time | STROOP 4 inhibition / switching (time) |
| Stroop1 2avg | STROOP mean time 1 and 2 |
| Stroop3 minus1 2avg | STROOP 3 minus mean time of STROOP1 and 2 |
| Stroop4 minus1 2avg | STROOP 4 minus mean time of STROOP1 and 2 |
| CP tap right | CP – Right finger tap motor speed |
| CP tap left | CP – Left finger tap motor speed |
| CP fas fon flow | CP – FAS Phonological flow |
| CP fas sem flow | CP – FAS Semantic flow |
| CP wm forward ls | CP – Visual working memory forward longest sequence completed |
| CP wm forward ss | CP – Visual working memory forward sum score |
| CP wm backward ls | CP – Visual working memory backward longest sequence completed |
| CP wm backward ss | CP – Visual working memory backward sum score |
| CP wm ss | CP – Visual working memory sum score |
| CP stroop congruent rt | CP – Spatial stroop congruent reaction time (ms) |
| CP stroop incongruent rt | CP – Spatial stroop incongruent reaction time (ms) |
| CP stroop errors | CP – Spatial stroop Errors |
| CP stroop num of reps | CP – Spatial stroop number of responses |
| CP stroop diff incong cong | CP – Spatial stroop incongruent minus congruent (ms) |
| CP spatspan ls | CP – Visual spatial span longest sequence competed |
| CP spatspan tot | CP – Visual Spatial span total |
| CP coding corr | CP – Coding number of correct responses |
| TVA K | TVA – Short-term memory storage (K) |
| TVA C | TVA – Processing speed (C) |
| TVA t0 | TVA – Perceptual threshold (*t_0_*) |
| TVA ErrorRate | TVA – Error rate |

Table S2. List of abbreviations used in Figure 4, 5 and S1. WASI= Wechsler Abbreviated Scale of Intelligence, CLVT= California Verbal Learning Test, STROOP= Delis-Kaplan Executive Function System (D-KEFS) color word interference test, CP= Cognitive Assessment at Bedside for iPAD (CabPAD), TVA= Theory of Visual Attention.
