# Supplementary figures and images for: Assessing distinct patterns of cognitive aging using tissue-specific brain age prediction based on diffusion tensor imaging and brain morphometry
Source: PeerJ. 2018 Nov 30;6:e5908. doi: 10.7717/peerj.5908 (PMC6276592; doi:10.7717/peerj.5908)

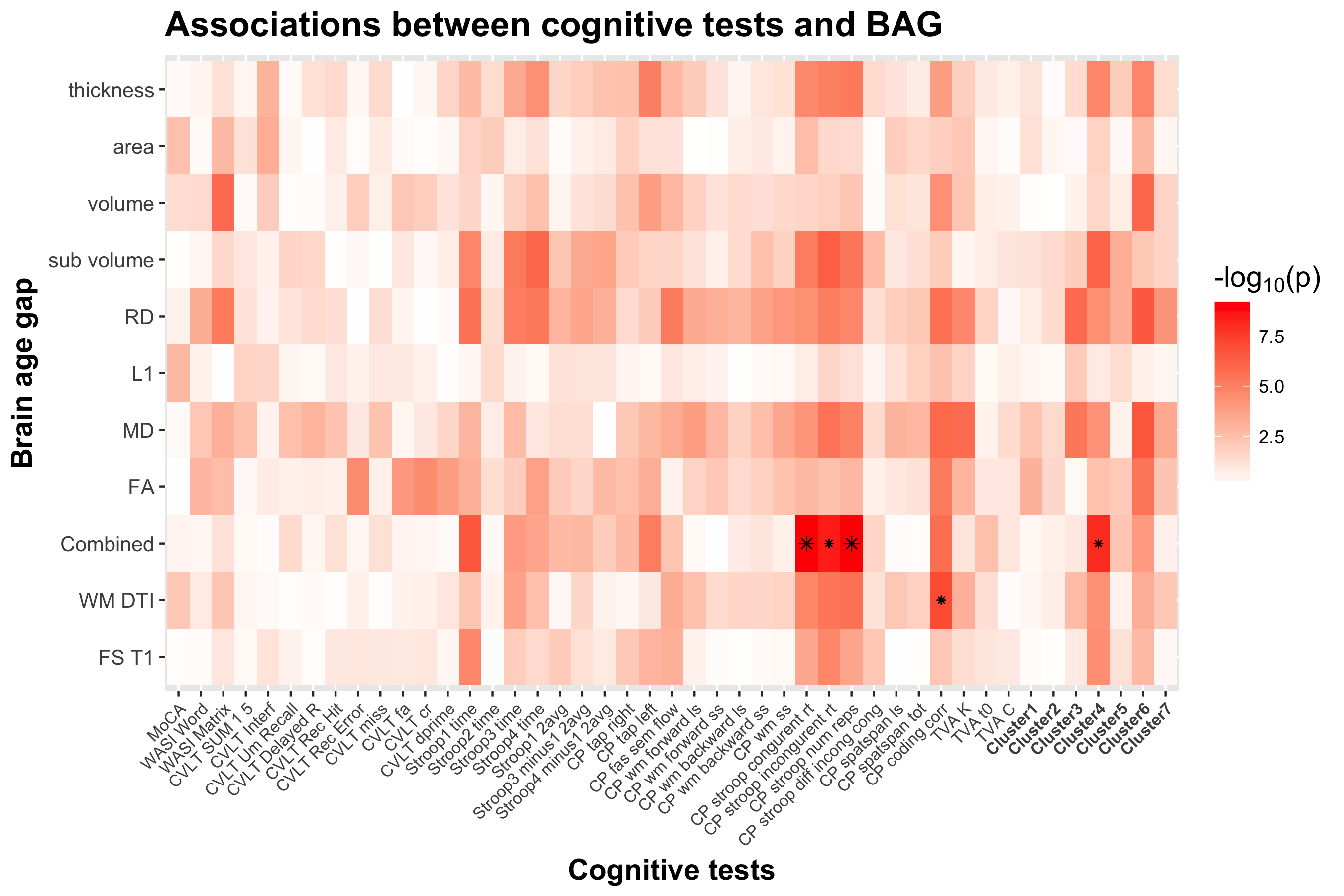

Supplement: Figure S1 — The color scale depicts the minus log of the p-values (−log10(p)) for each association. The association marked with a small star represents significant associations after FDR correction, and the one marked with a big star shows significant associations after Bonferroni correction. Suppl. table S2 provides detailed overview of all abbreviations used. [file peerj-06-5908-s003.png]
